# Flexible suction–coagulation probe restores dexterity in robot-assisted surgery: bench-to-bedside evaluation

**DOI:** 10.1007/s00464-025-12138-w

**Published:** 2025-09-10

**Authors:** Yuki Ushimaru, Shohei Shibamoto, Kazuyoshi Yamamoto, Yoshitomo Yanagimoto, Namiko Ban, Nobuaki Tagi, Makoto Hosaka, Naoto Gotohda, Hidetoshi Eguchi, Yuichiro Doki, Kiyokazu Nakajima

**Affiliations:** 1 Department of Next Generation Endoscopic Intervention (Project ENGINE), Graduate School of Medicine, The University of Osaka, Suite 0802, BioSystems Bldg., 1-3, Yamadaoka, Suita, Osaka 565-0871 Japan; 2 Department of Gastroenterological Surgery, Osaka International Cancer Institute, Osaka, Japan; 3 Department of Gastroenterological Surgery, Graduate School of Medicine, The University of Osaka, Osaka, Japan; 4 Yamashina Seiki Co. Ltd., Ritto, Shiga Japan; 5 AMCO Incorporated, Tokyo, Japan; 6 Department of Hepato-Biliary-Pancreatic Surgery, National Cancer Center Hospital East, Kashiwa, Japan

**Keywords:** Robotic surgical procedures, Electrosurgery, Hemostasis, Laparoscopy, Equipment design

## Abstract

**Objective:**

Rigid suction–coagulation probes constrain the wrist-like articulation that is central to robotic surgery. We therefore designed a 5-mm single-use flexible suction ball coagulator (flex-SBC) with a modified core design to restore dexterity and assessed its mechanical performance and early clinical feasibility, including the effect of the common robotic gripping strategies on suction flow.

**Methods:**

Preclinical. The new 7 × 7 core embedded in silicone was compared with the conventional 1 × 7 core design. Shaft pliability was quantified by sagging displacement and rebound force testing. Suction flow at − 20, − 30, and − 40 kPa was measured, and the impact of grip location (electrode base vs silicone shaft) and forceps type (fenestrated or Maryland bipolar) was analyzed statistically. Clinical. The flex-SBC was used in 12 consecutive robotic gastrectomies with prospective collection of device performance, adverse events, and surgeon rating on seven-domain via three-point Likert scale.

**Results:**

Preclinical. Adopting the 7 × 7 increased mean sagging displacement from 15.6 ± 1.8 to 39.3 ± 3.2 mm (*p* < 0.001) and reduced rebound force from 0.288 ± 0.014 to 0.059 ± 0.004 N (*p* < 0.001). Mean flow rates were 6.76 ± 0.88, 8.87 ± 0.43, and 10.58 ± 0.40 mL/sec at − 20, − 30, and − 40 kPa, respectively—approximately half those of a rigid probe (all *p* < 0.001) but still exceeding published thresholds for effective evacuation. Gripping the silicone shaft, especially with Maryland forceps, sharply reduced flow (60% reduction; *p* < 0.0011). Clinical. All operations were completed without device-related malfunctions, injuries, or conversions. Of 84 survey ratings, connection setup, suction efficiency, tissue safety, and overall satisfaction were “satisfactory” in 100%; maneuverability was “satisfactory” in 65% and “average” in 35%. No “unsatisfactory” scores were recorded.

**Conclusions:**

The flex-SBC bends where the robot bends, enabling safe, practical use across robot-assisted upper-GI surgery. Grip technique influences device performance, providing important implications training. Larger comparative trials should clarify whether these ergonomic gains translate into shorter operating times and improved oncologic precision.

**Supplementary Information:**

The online version contains supplementary material available at 10.1007/s00464-025-12138-w.

Very-low-voltage electrocoagulation has been shown to deliver reliable hemostasis with minimal thermal spread in both bench and clinical settings [1]. When the same energy is delivered through a probe that integrates suction, it can simultaneously evacuate blood and surgical plume, reducing instrument exchanges and further limiting collateral injury [1–6]. Currently available suction–coagulation probes are rigid with high flexural stiffness; when inserted into the curved working envelope of robotic instruments, this stiffness resists intended angulation, causing the shaft to “bend back” from the target site and even push against the robotic arms, making it difficult or impossible to reach certain anatomical areas. Conversely, if made too flexible, the shaft risks collapsing under negative pressure during suction. An optimal balance of stiffness is therefore essential—sufficient to maintain lumen patency under aspiration while flexible enough to conform to the robot’s articulation and remain at the target without recoil. This trade-off is critical for enabling the probe to function as a stable, hands-free sump and for meeting the 6–8 mL/sec (360–480 mL/min) flow rates required to keep the upper abdomen clear during major resections [7–9]. Moreover, hand-offs to the assistant interrupt bimanual control during critical steps such as D2 lymphadenectomy and bleeding control [10, 11].

To address these limitations, we collaborated within a multidisciplinary device-development team to create a single-use flexible suction–ball coagulation probe (flex-SBC, SBC: suction ball coagulator) in our consortium [12]. The probe embeds a 7 × 7 stranded stainless steel core in medical-grade silicone shaft, allowing it’s flexible core to “bend where the robot steerable instrument of the bends” while preserving electrical conductivity and lumen patency. Although optimized for articulated robotic instruments, the device is compatible with standard 5 mm trocars, making it usable in straight-stick laparoscopy and hybrid approaches. Robotic gastrectomy, particularly in gastric cancer treatment, now demonstrates oncologic non-inferiority and, in some series, reduced morbidity with faster return to adjuvant therapy [13–15], driving rapid global uptake thanks to 3D optics, tremor filtration, and wrist-like articulation [16].

Accordingly, the present study (i) quantifies how the new 7 × 7-stand core shaft improves pliability and rebound force compared with the legacy 1 × 7 core design, (ii) characterizes pressure-dependent flow and the impact of typical robotic gripping strategies on a lumen patency, and (iii) demonstrates first-in-human feasibility of the flex-SBC as a drop-in sump that delivers very-low-voltage electrocoagulation while keeping the upper-abdominal field clear during complex robotic-assisted gastrectomies.

## Materials and methods

### Device design and development

**Fig. 1:**
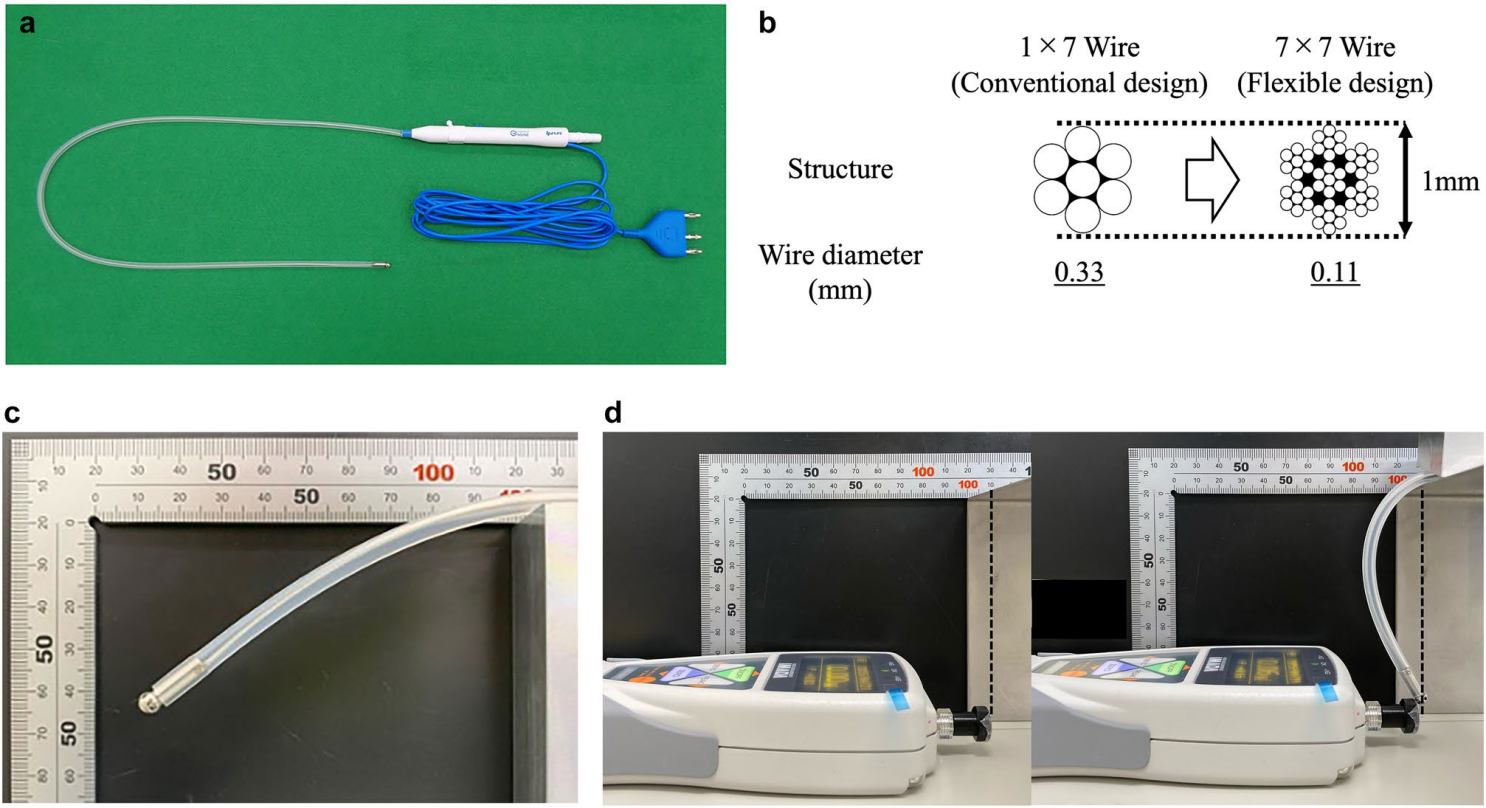
Mechanical design and flexibility testing of the flexible suction–coagulation probe. **a** Flexible suction–coagulation probe consisting of a flexible silicone shaft reinforced with a 7 × 7 stranded stainless steel wire. **b** Cross-sectional schematic of the conductive core: the new probe incorporates a 7 × 7 stranded stainless-steel bundle, whereas the legacy design used a 1 × 7 configuration. **c** Sagging displacement setup. With 100 mm of shaft extending horizontally beyond a support block, vertical tip deflection under gravity was recorded against a right-angle ruler (three repetitions). **d** Rebound force measurement. In the same horizontal position, the probe tip was gently compressed against a digital force gauge; the peak reactive force was taken as the rebound value (three repetitions)

The flex-SBC was developed by modifying the structure of the conventional rigid SBC (Fig. 1a). A 7 × 7 stranded stainless steel wire was embedded within a medical-grade silicone tube to serve as the conductive core, thereby improving shaft flexibility while maintaining electrical conductivity (Fig. 1b). The ball-shaped electrode tip, with a diameter of 5.0 mm, remained consistent with the original SBC design [2, 3]. The total shaft length was set at 700 mm to accommodate the working environment of robotic-assisted laparoscopic surgery.

### Preclinical testings

#### Flexibility testing

To assess flexibility, two types of mechanical tests were performed: sagging displacement and rebound force measurement (Fig. 1c). For the sagging test, a right-angle ruler (TK-503CN, Trusco Nakayama Corporation, Japan) was fixed vertically to a wall (Fig. 1).

The flex-SBC was positioned such that 100 mm of the shaft extended horizontally beyond a supporting block, and the vertical displacement of the tip due to gravity was measured. Three independent measurements were performed, and the average sagging displacement was calculated.

For the rebound force test, elasticity was evaluated using a force gauge (ZTA-50N, Imada Co., Ltd., Japan) (Fig. 1d). In the same horizontal extension setup, the tip of the SBC was gently pressed against the force gauge sensor until measurable deformation occurred, and the maximum reactive force was recorded. Both tests were conducted under standardized room conditions. These mechanical characterizations were intentionally performed separately from suction flow measurements to isolate intrinsic shaft pliability and recoil behavior without confounding fluid dynamics; this separation establishes a structural baseline before assessing functional suction performance.

#### Suction performance testing

**Fig. 2:**
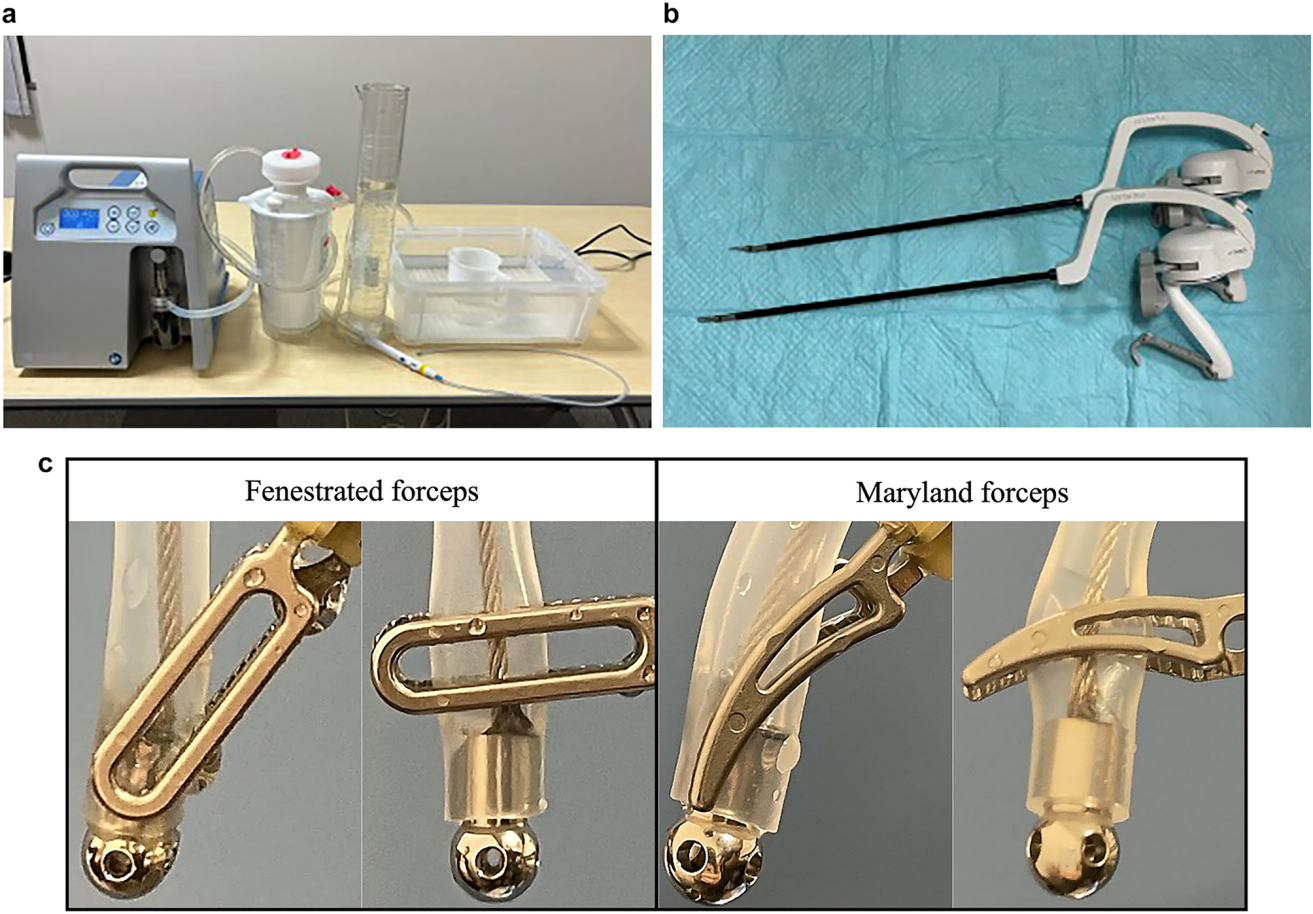
Benchtop flow assessment and gripping configurations for the flexible suction–coagulation probe. **a** Experimental layout for water-suction testing: the probe is connected to a commercial suction unit and immersed in a 1 L graduated cylinder filled with room-temperature water; negative pressure (− 20, − 30, or − 40 kPa) is supplied through the central lumen, and the time to evacuate each 50 mL increment is recorded. **b** Artisential® robotic forceps used in the study; fenestrated bipolar, Maryland bipolar. **c** Two gripping strategies evaluated at − 40 kPa. Left panels: electrode-base grasping (metal tip held); right panels: shaft grasping (silicone segment held). Upper row, fenestrated bipolar; lower row, Maryland bipolar

Suction performance was assessed using a controlled benchtop setup (Fig. 2). A total of 1000 mL of tap water at room temperature (22–24 °C) was placed in a graduated cylinder. The flex-SBC was connected to a commercial suction unit (MV2-1400, Shin-Ei Industries Inc., Tokyo, Japan) and suction was tested under three negative pressure settings: 20, 30, and 40 kPa. The time required to aspirate each 50 mL increment was recorded using a calibrated stopwatch.

Suction speed (mL/sec) was calculated based on the aspirated volume and time, and suction efficiency (mL/sec/kPa) was obtained by normalizing suction speed to the applied pressure. Each measurement was performed in triplicate to ensure reliability.

#### Influence of gripping location and forceps type

The effects of gripping conditions were also evaluated. Two gripping sites were compared: the electrode base (proximal to the ball electrode) and the shaft tube section.

Multi-joint bipolar fenestrated and Maryland forceps (Artisential®, LivsMed Inc., Republic of Korea) were used only in these preclinical grasping experiments to assess how grip location and jaw configuration affect lumen patency and flow; these instruments were not employed intraoperatively.Each gripping-forceps combination was tested three times under a suction pressure of 40 kPa.

### Clinical evaluation

The flexible SBC is certified as a Class II medical device in Japan (certification number 226AFBZX00158000) and, with written informed consent, was used in twelve robotic-assisted gastrectomies for gastric or gastro-oesophageal-junction cancer. Intraoperative observations documented suction performance, coagulation efficacy and instrument handling. Immediately after each procedure, the console surgeon completed a seven-domain, a three-point Likert questionnaire (3 = satisfactory, 2 = average, 1 = unsatisfactory) covering seven domains: connection setup, trocar insertion, maneuverability, suction efficiency, coagulation efficiency, tissue safety, and overall satisfaction. All operations were performed with the da Vinci Xi system (Intuitive Surgical, Sunnyvale CA, USA).

### Statistical analysis

Continuous data are presented as mean ± standard deviation. Two-group comparisons—for wire configurations, for each negative pressure setting, and for each pair of grip positions—were performed with the Wilcoxon rank-sum test. Suction speeds across the three pressure settings were first analyzed with the Kruskal–Wallis test; when significant, pairwise differences were probed with Wilcoxon tests. Analyses were executed in JMP Pro 18.2.0 (SAS Institute, Cary, NC, USA). A two-sided *p*-value below 0.05 was considered statistically significant.

## Results

### Preclinical testings

#### Flexibility testing

The modified 7 × 7-strand conductive core produced a marked increase in shaft pliability (Table 1). Mean sagging displacement almost tripled relative to the conventional 1 × 7 design (39.3 ± 3.2 mm vs. 15.6 ± 1.8 mm; *p* = 0.0008), while rebound force fell to roughly one-fifth of its original value (0.059 ± 0.004 N vs. 0.288 ± 0.014 N; *p* = 0.0005).

**Table 1:** Flexibility comparison between wire types

Parameter	7 × 7 wire (flexible design)	1 × 7 wire (conventional design)	*p*-Value
Stranded wire specifications	Wire diameter *φ* 0.33 × 7	Wire diameter *φ* 0.11 × 49	–
Sagging (mm)	39.3 ± 3.2	15.6 ± 1.8	0.0008
Rebound force (*N*)	0.059 ± 0.004	0.288 ± 0.014	0.0005

#### Suction performance testing

##### Pressure-dependent flow characteristics

**Fig. 3:**
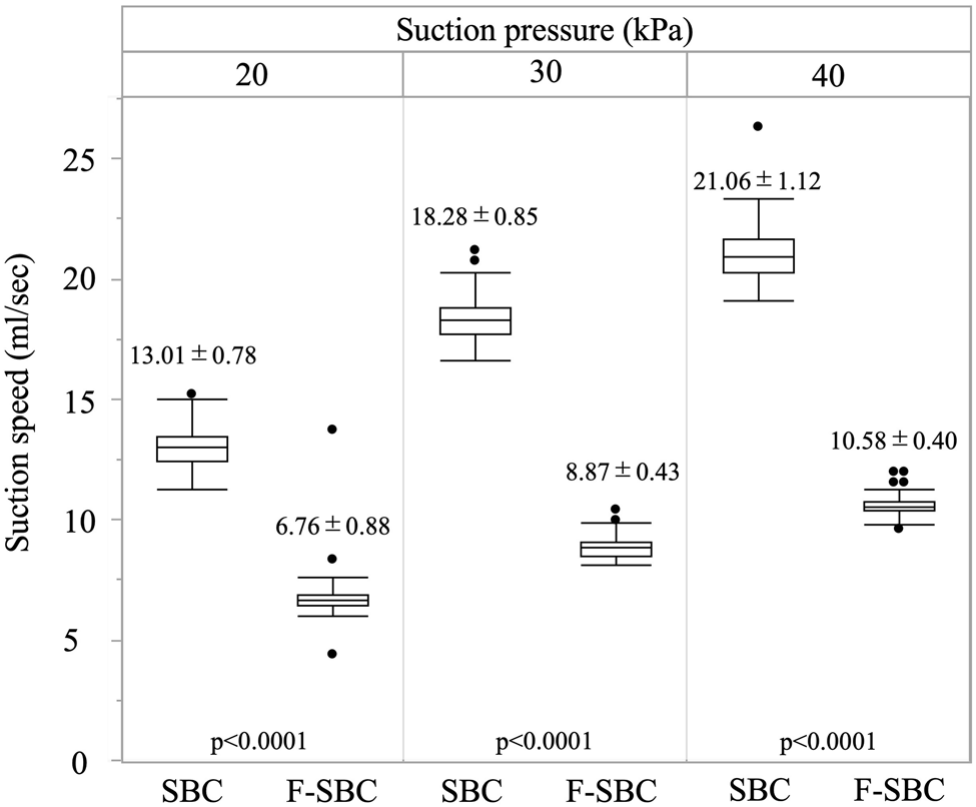
Suction speed at three vacuum levels. Box-and-whisker plots compare suction speed (mL/sec) of the conventional rigid suction ball coagulator (SBC) and the flexible suction ball coagulator (F-SBC; “flex-SBC”) at three negative pressure settings (− 20, − 30, and − 40 kPa). Boxes indicate the inter-quartile range, the horizontal line represents the median, whiskers depict the full data range (*n* = 5 trials per device per pressure), and solid circles denote individual measurements outside the IQR. Mean ± SD values are printed above each box. Paired comparisons between devices at each pressure were performed with two-tailed t-tests; all were highly significant (*p* < 0.0001)

Across all negative pressure settings, the flex-SBC aspirated fluid more slowly than the rigid laparoscopic model (Fig. 3). At − 20, − 30, and − 40 kPa, mean flow rates were 6.76 ± 0.88, 8.87 ± 0.43, and 10.58 ± 0.40 mL/s, respectively—approximately 50% of the conventional device (*p* < 0.0001 for each comparison).

##### Influence of gripping location and forceps type

**Fig. 4:**
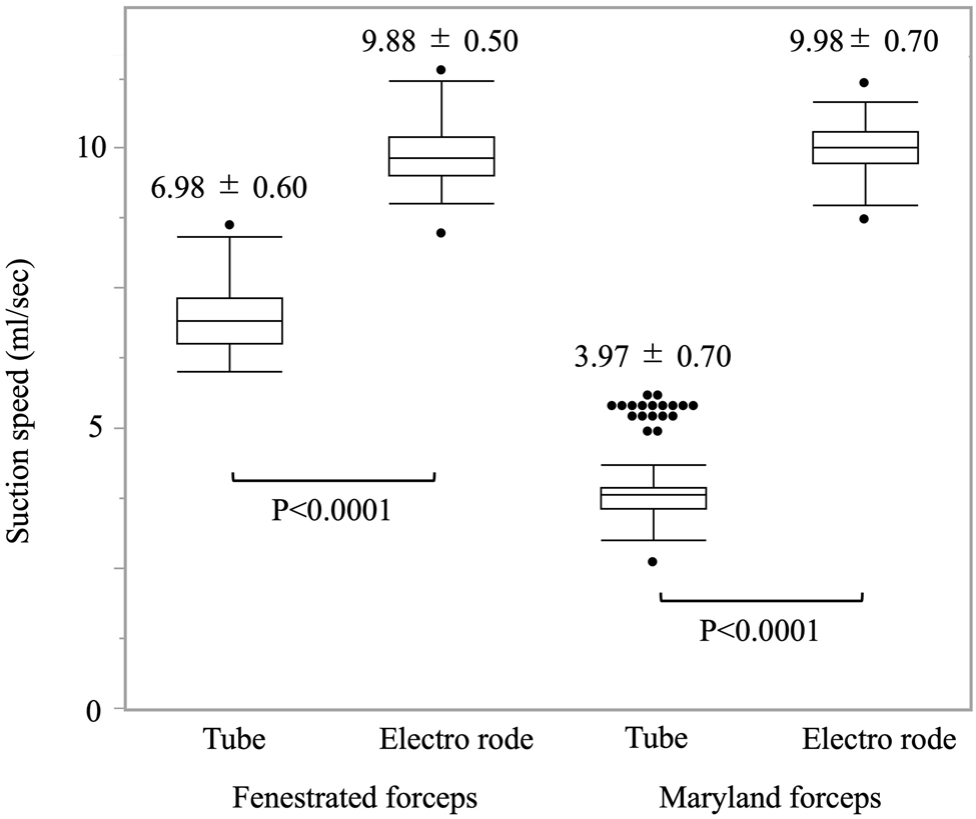
Suction speed by grip site and forceps. Flow at − 40 kPa when the probe is held at the metal tip (“Base”) or the silicone shaft (“Tube”) with Fenestrated or Maryland bipolar jaws

Suction efficiency varied significantly with the site of instrument grasp and the type of robotic forceps employed (Fig. 4). When the electrode base was held, flow remained high, whereas grasping the silicone tube immediately proximal to the metallic probe caused a pronounced decline. With fenestrated bipolar forceps, flow fell from 9.88 ± 0.50 mL/sec (electrode-base grip) to 6.98 ± 0.60 mL/sec (tube grip; *p* < 0.0001). The greatest reduction—approximately 60%—was observed with Maryland bipolar forceps (9.98 ± 0.70 mL/sec vs. 3.97 ± 0.70 mL/sec; *p* < 0.0001).

### Clinical evaluation

**Fig. 5:**
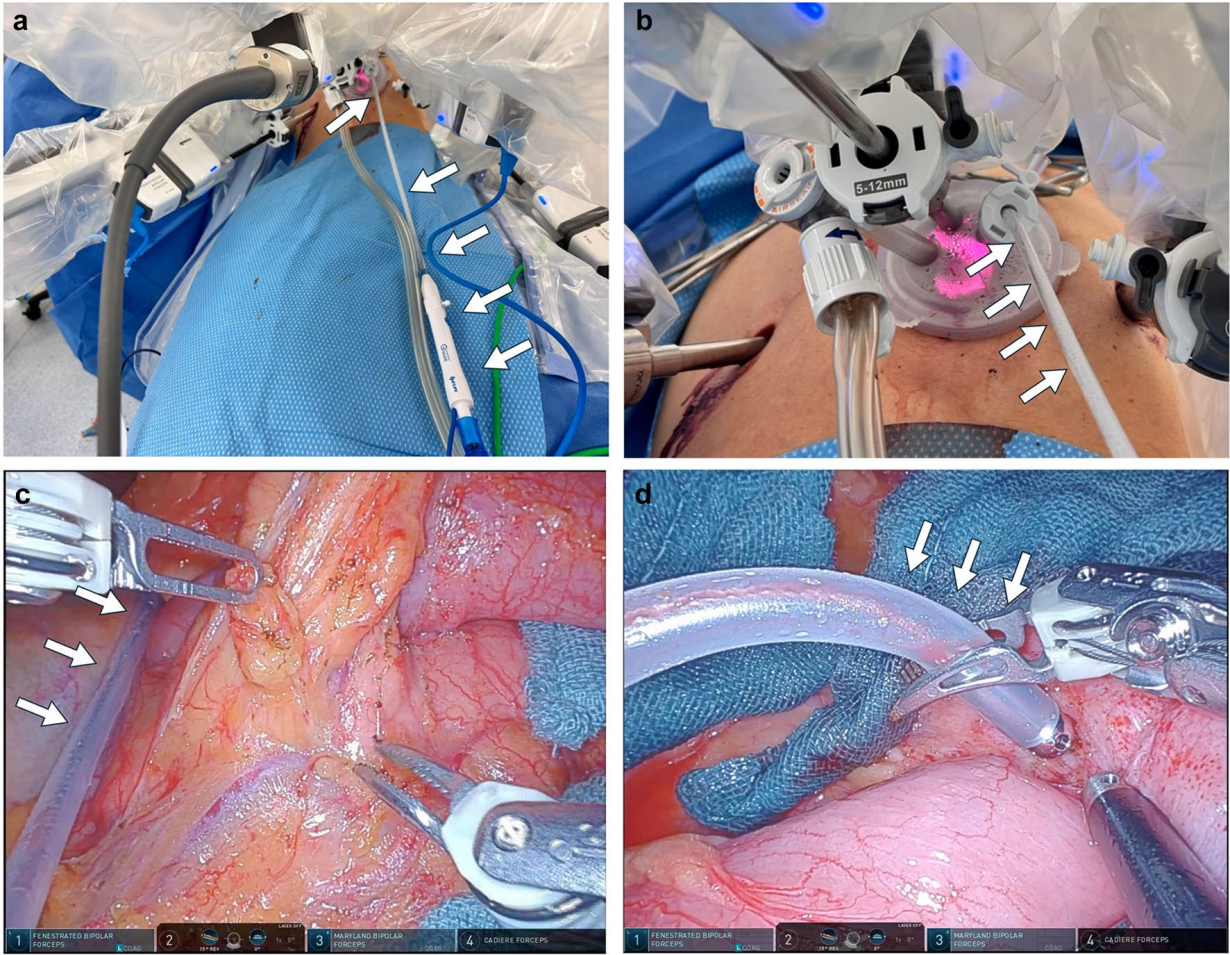
Insertion and use of the flex-SBC. **a** External wide view of the flex-SBC being introduced into an additional 5 mm trocar: The device is shown from outside the patient, demonstrating the entry trajectory alongside the robotic setup. White arrows indicate the flex-SBC. **b** External close-up view of the flex-SBC shaft during insertion through the 5 mm trocar, highlighting the flexible segment advancing while preserving the working envelope of the robotic instruments. White arrows indicate the flex-SBC. **c** Intraoperative resting position (“dropped-in” sump): The flex-SBC is left in place to provide continuous hands-free suction, including evacuation of pooled fluid. White arrows indicate the flex-SBC. **d** Active hemostasis: The flex-SBC enables simultaneous suction and coagulation while the console surgeon exploits full instrument articulation to control bleeding with a clear field. White arrows indicate the flex-SBC

**Fig. 6:**
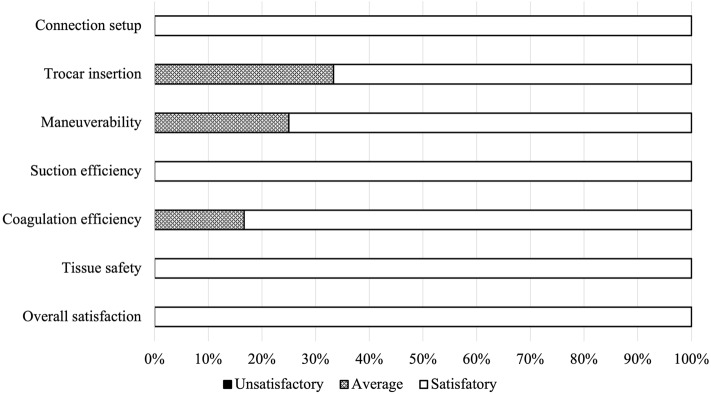
Post-operative surgeon survey of the flexible suction–coagulation probe. Distribution of responses for seven domains (*n* = 10 procedures) on a three-point Likert scale: 3 = satisfactory, 2 = average, 1 = unsatisfactory

Twelve patients (seven men, five women; median age 68 years, range 27–87) underwent flex-SBC–assisted robotic gastrectomy—nine for gastric cancer and three for gastro-oesophageal junction cancer (Supplemental Video) (Fig. 5). The series comprised five robotic distal gastrectomies, four total gastrectomies and three extended proximal gastrectomies. No device-related failures, malfunctions or complications occurred. Surgeons completed a seven-domain, three-point Likert survey immediately post-procedure (84 responses total) (Fig. 6). Connection setup, suction efficiency, tissue safety, and overall satisfaction each received a “satisfactory” rating in 100% of responses. Trocar insertion and coagulation efficiency were rated “satisfactory” in 70% and “average” in 30%, while maneuverability was rated “satisfactory” in 65% and “average” in 35%. No “unsatisfactory” ratings were recorded in any domain, and no tissue injury attributable to the flex-SBC was observed.

## Discussion

To overcome the constrains of working with straight suction combined with coagulation, we developed a single-use, 5-mm flexible suction ball coagulator (flex-SBC). The observations in our study showed that the 7 × 7 stranded stainless steel core embedded in silicone allows the shaft to “snake” around fixed obstacles so the electrode tip reaches bleeding points along curved or oblique trajectories that are impossible with conventional probes [17–19]. Once the tip is positioned, continuous negative pressure creates a hands-free sump, preserving optical clarity while the console surgeon continues bimanual dissection [1, 4–6].

Bench testing confirmed that flexibility is gained without catastrophic loss of flow. Flexibility testing was conducted independently from suction flow measurements to quantify intrinsic shaft pliability and recoil without confounding effects from fluid dynamics; the shaft material was selected to maintain lumen patency even under moderate bending, so flow was not expected to be substantially reduced by curvature. Even at − 40 kPa, the mean suction speed remained > 10 mL/sec—well above the 6–8 mL/sec considered adequate for blood evacuation in laparoscopic hepatectomy [7–9]. Although the flex-SBC’s mean suction speed remains approximately half that of a rigid probe, it exceeds typical intraoperative bleeding rates (rarely > 100 mL/min, about 1.7 mL/sec) and thus provides sufficient flow to manage high-flow bleeding scenarios while restoring instrument dexterity. Gripping experiments showed that clamping the electrode base with fenestrated bipolar forceps maintains lumen patency, whereas grasping the silicone shaft or using narrow Maryland jaws markedly reduces flow, providing clear ergonomic guidance for robotic training programs [17–19].

Clinical feasibility was demonstrated in ten complex upper-abdominal and hepatic resections. Although no formal coagulation bench study was included, surgeons uniformly rated hemostasis as “satisfactory,” mirroring the efficacy of soft coagulation energy combined with suction reported in porcine and clinical studies [1, 4–6]. Although our series did not include formal hemostasis timing, previous prototyping and clinical use of the SBC demonstrated prompt coagulation with no device-related adverse events in 17 laparoscopic colorectal surgeries [3]. Furthermore, bench and preclinical studies of very-low-voltage soft coagulation with simultaneous suction showed success rates exceeding 95% and minimal tissue adherence [1], supporting the flex-SBC’s effective coagulation performance. The device fitted all existing 5 mm ports and required no additional capital investment. By functioning as a continuous, hands-free sump, the flex-SBC reduces reliance on assistant-held suction, preserves console surgeon bimanual autonomy, and minimizes instrument exchanges—thereby streamlining workflow during critical phases such as bleeding control and D2 lymphadenectomy. When not actively coagulating, the flex-SBC tip can be placed in natural recesses to deliver low-level continuous suction, keeping the field dry; in cases of sudden bleeding, its presence eliminates instrument exchanges and preserves surgical flow. Although adjustments to suction strength still require bedside assistance, the device empowers the console surgeon to manage both suction and coagulation autonomously, even when communication with the patient-side assistant is delayed.

Several limitations warrant mention. Suction tests employed water rather than whole blood, which is more viscous and particulate; real-world flow may therefore be lower. Only ten clinical cases were evaluated and no rigid probe control arm was included, precluding quantitative assessment of time savings or complication reduction. This feasibility series lacks a formal control arm and did not include quantitative coagulation bench testing or intraoperative flow measurement. The Artisential® instruments depicted were used exclusively in preclinical experiments; clinical cases employed only the da Vinci Xi system, and the preclinical setup may not fully recapitulate intraoperative conditions. Finally, we did not measure thermal spread, burst pressure, or tissue stickiness—metrics that should be addressed in future ex vivo liver and survival animal models. These findings suggest that handling recommendations—such as favoring electrode-base grasping—should be included in surgeon orientation materials and device instructions to help maintain lumen patency and optimize suction performance. The flex-SBC is intended as an adjunct accessory, compatible with both straight-stick laparoscopy and articulated robotic platforms via standard forceps. While the present version relies on manual forceps actuation for suction and coagulation, future iterations may explore a fully robotic end-effector design to automate these functions. Additionally, this feasibility series did not include formal ex vivo coagulation assessments—such as quantification of thermal spread and sealing reliability—but we plan dedicated laboratory studies in porcine liver models to evaluate these parameters in future work.

In conclusion, flex-SBC restores robotic dexterity to suction–coagulation tasks. By bending where the robot bends, maintaining clinically useful flow and enabling hands-free sump suction, it keeps the field dry and stable during the most technically exacting steps of gastric and hepatic surgery. Larger comparative trials and dedicated hemostasis testing will determine whether these ergonomic gains translate into shorter operative times, enhanced lymph-node clearance and, ultimately, improved long-term oncologic outcomes.

## Supplementary Information

Below is the link to the electronic supplementary material.

Supplementary file1 (MP4 142743 KB)
